# Comparison of analgesic interventions for traumatic rib fractures: a systematic review and meta-analysis

**DOI:** 10.1007/s00068-018-0918-7

**Published:** 2018-02-06

**Authors:** Jesse Peek, Diederik P. J. Smeeing, Falco Hietbrink, Roderick M. Houwert, Marije Marsman, Mirjam B. de Jong

**Affiliations:** 1 Department of Surgery, University Medical Center Utrecht, Utrecht, The Netherlands; 2 Utrecht Traumacenter, Utrecht, The Netherlands; 3 Department of Anesthesiology, University Medical Center Utrecht, Utrecht, The Netherlands

**Keywords:** Analgesia, Anesthesia, Hospitalization, Mortality, Pain Management, Rib Fractures

## Abstract

**Purpose:**

Many studies report on outcomes of analgesic therapy for (suspected) traumatic rib fractures. However, the literature is inconclusive and diverse regarding the management of pain and its effect on pain relief and associated complications. This systematic review and meta-analysis summarizes and compares reduction of pain for the different treatment modalities and as secondary outcome mortality during hospitalization, length of mechanical ventilation, length of hospital stay, length of intensive care unit stay (ICU) and complications such as respiratory, cardiovascular, and/or analgesia-related complications, for four different types of analgesic therapy: epidural analgesia, intravenous analgesia, paravertebral blocks and intercostal blocks.

**Methods:**

PubMed, EMBASE and CENTRAL databases were searched to identify comparative studies investigating epidural, intravenous, paravertebral and intercostal interventions for traumatic rib fractures, without restriction for study type. The search strategy included keywords and MeSH or Emtree terms relating blunt chest trauma (including rib fractures), analgesic interventions, pain management and complications.

**Results:**

A total of 19 papers met our inclusion criteria and were finally included in this systematic review. Significant differences were found in favor of epidural analgesia for the reduction of pain. No significant differences were observed between epidural analgesia, intravenous analgesia, paravertebral blocks and intercostal blocks, for the secondary outcomes.

**Conclusions:**

Results of this study show that epidural analgesia provides better pain relief than the other modalities. No differences were observed for secondary endpoints like length of ICU stay, length of mechanical ventilation or pulmonary complications. However, the quality of the available evidence is low, and therefore, preclude strong recommendations.

## Introduction

Traumatic rib fractures are a common injury among the trauma population and can cause severe pain in both isolated rib fractures and fractures which are a part of more extensive chest injuries [1, 2]. Rib fractures are clinically important. Even isolated fractures are associated with significant consequences, such as prolonged pain and disabilities [3]. Rib fractures sustained following blunt chest trauma are a surrogate for significant trauma, particularly in more vulnerable patients [1, 4, 5]. The number of rib fractures is indicative of the trauma severity. More than 90% of the patients with multiple rib fractures have associated injuries, most commonly involving head, abdomen and/or extremities [1]. An increased number of fractures, older age, and polytrauma patients with rib fractures are associated with increased rates of morbidity and mortality [1, 4, 5].

The thoracic pain caused by rib fractures or chest contusion limits patients to cough and breathe deeply, which can result in atelectasis and pneumonia. Besides most of these, patients also suffer from a pulmonary contusion, due to their injury. This can lead to an acute respiratory distress syndrome and/or respiratory failure and the need for mechanical ventilation has been reported [6, 7].

A combination of adequate pain control, respiratory assistance, and physiotherapy are considered to be the key in the management of patients with fractured ribs [4, 8]. In the current practice, different analgesic modalities including epidural catheters, intravenous (patient controlled) narcotics, intercostal, paravertebral or interpleural blocks, oral opioids, or a combination of the aforementioned interventions, are used as therapy [9, 10].

The literature on the use of the different analgesic interventions is inconclusive. A clinical guideline supported by the Eastern Association for the Surgery of Trauma recommends epidural analgesia or a multimodal approach over opioids alone in patients with blunt chest trauma [9]. On the other hand, two recently performed systematic reviews and meta-analyses of Duch et al. [10] and Carrier et al. [11] stated that the evidence for the use of epidural analgesia as preferred modality is insufficient, and that there is no firm evidence for benefit or harm of the epidural modality compared to the other interventions.

However, to date, no comprehensive study compared the single modalities independently with each other, including both observational studies and randomized controlled trials. Therefore, the aim of this systematic review and meta-analysis is to compare epidural, intravenous, paravertebral and intercostal analgesia for the primary outcome of pain reduction and the secondary outcomes of mortality during hospitalization, length of mechanical ventilation, length of hospital stay, length of intensive care unit stay (ICU) and complications, in patients with traumatic rib fractures.

## Methods

A published protocol for this review does not exist. No ethical committee approval was necessary for this literature review.

### Literature search and eligibility criteria

This systematic review and meta-analysis was written in accordance to the PRISMA guidelines for reporting systematic reviews and meta-analyses [12]. Two reviewers (JP, DS) independently performed a structured literature search, on September 16th 2017, to identify comparative studies investigating epidural, intravenous, paravertebral and intercostal interventions for blunt chest trauma with traumatic rib fractures. Three different electronic databases (PubMed, EMBASE and CENTRAL) were used to perform a systematic search. The search strategy included keywords and MeSH or Emtree terms relating to traumatic rib fractures, analgesic interventions, pain management and complications. The full search syntax is provided in Appendix Table 2. The search was not restricted by date or any other limits.

**Fig. 1:**
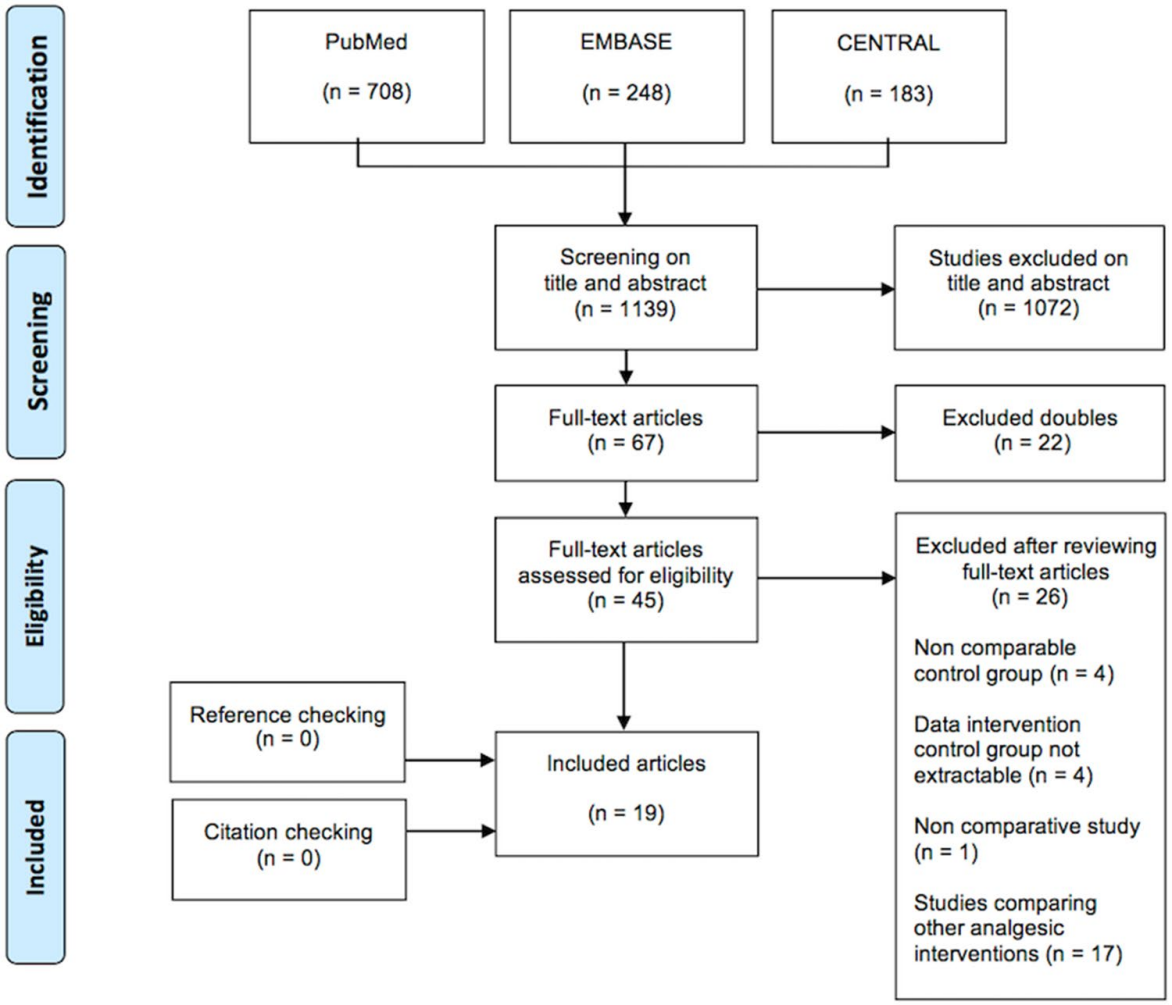
PRISMA flow diagram representing the search and screen process of articles describing analgesic interventions in patients with traumatic rib fractures

After screening of all titles and abstracts of the identified studied, full texts were obtained of the remaining relevant studies. Two reviewers (JP, DS) read the full-text articles, removed duplicates and made a final selection of relevant studies. Reference lists of retrieved articles were checked and citation tracking was performed using Web of Science, to identify articles not found in the original search. Figure 1 shows a flowchart of the search strategy.

Manuscripts were eligible for inclusion if published in English, French or Dutch language and available in full-text. Studies describing mixed cohorts of patients with blunt chest trauma, including traumatic rib fractures, were also eligible for inclusion. Animal studies, abstracts for conferences, studies including patients below 16 years of age, case reports and studies with less than five patients were excluded. There were no further restrictions for inclusion.

Authors were approached if additional information was needed or if full-text was not available.

### Quality assessment

The methodological quality of the articles was independently assessed by two reviewers (JP, DS) using the validated methodological index for non-randomized studies (MINORS) score [13]. Additional criteria, described in Appendix Table 3, were defined to make further distinction in quality between the included studies. The quality was determined by means of the total MINORS score. Studies were not excluded based on the quality assessment. Disagreement was resolved by discussion with a third independent reviewer (MJ), followed by consensus.

### Data extraction

Data were retrieved by two independent reviewers (JP, DS). Data extracted included first author, year of publication, country, study design, setting and treatment groups. For each treatment group, age, sex, type of analgesia and injury severity score (ISS) were extracted. The extracted data were shown as mentioned in the original studies. If exact pain scores were not given, an estimation of the scores was made on the basis of the figures. Outcomes were retrieved including confidence intervals (CI’s) and/or *p* values.

### Outcome measures

The predefined primary outcome was the reduction of pain, preferably expressed in a Numeric Rating Scale (NRS). Secondary outcomes were mortality during hospitalization, length of mechanical ventilation, length of hospital stay, length of intensive care unit stay (ICU) and complications.

### Data analysis

Data were pooled according to the analgesic modalities that were compared. Meta-analyses were performed if the endpoints were reported by two or more studies. If the extracted data were initially noted as median with an interquartile range, the mean and standard deviation (SD) were estimated as follows: the reported median value was used as mean value, and the standard deviation was estimated by dividing the interquartile range with 1.35. Statistical heterogeneity was assessed by visual inspection of the forest plots and estimated by means of the *I*^2^, Tau^2^ and Cochran’s Q (Chi-square test). A random-effects model was used if high heterogeneity was present (where *I*^2^ > 75% reflects a high heterogeneity). Odds ratios and 95% confidence intervals (95% CI) were calculated for dichotomous variables. Studies that reported zero events in one or both arms were included by adding a continuity correction of 1.0 to all cells in the 2 × 2 table of that study [14]. *p* values < 0.05 were considered statistically significant.

After the primary statistical analyses, sensitivity and subgroup analyses were conducted. In the sensitivity analyses on study design, only RCTs were included. In the sensitivity analyses on time, only studies published after the year 2000 were included. In the sensitivity analyses on quality, arbitrarily all studies with more than 16 points were included [15]. A sensitivity analyses on outlier studies was conducted. For the subgroup analyses on etiology, only studies describing cohorts with solely traumatic rib fractures were included. Studies describing mixed cohorts of patients with blunt chest trauma were excluded.

All statistical analyses were performed using Review Manager (RevMan, Version 5.3.5 Copenhagen: The Nordic Cochrane Centre, The Cochrane Collaboration, 2014).

## Results

### Search

The literature search yielded 1129 studies and after removal of duplicates and screening titles and abstracts for relevance, 44 articles were assessed for eligibility. After application of the inclusion and exclusion criteria, 19 articles were finally included in this systematic review [6, 8, 16–32]. Twenty-four studies were excluded, mainly because analgesic modalities, other than epidural, intravenous, paravertebral or intercostal were described [33–46]. Five studies were excluded because data of the interventions used in the control group could not be extracted [4, 47–50]. There were no eligible studies excluded by the language restriction. No additional articles were identified during the reference and citation check. A flow chart of the complete selection procedure is shown in Fig. 1.

### Quality assessment

The total MINORS score of the included articles are listed in Appendix Table 3. On average the included articles scored 15.7 ± 2.9 points, with a range of 11–23 points.

### Baseline characteristics

Of the 19 included studies, 8 were RCTs, 10 were retrospective cohort studies, and 1 study was a prospective cohort study using a historical control group. The included studies describe a total of 2801 patients. Eleven studies [8, 16–21, 27–29] compared epidural analgesia with intravenous analgesia. Eight of these studies [4, 16–18, 20, 21, 27, 28] compared epidurals with local anesthetics with or without opioids as drugs, with intravenous analgesia. Three studies [19, 24, 29] compared epidurals, with only opioids as drugs, with intravenous analgesia. Three studies [22, 25, 26] compared epidural analgesia with intercostal blocks, three studies compared epidural analgesia with paravertebral blocks [6, 30, 31], one study compared paravertebral blocks with intravenous analgesia [32] and one study [23] compared intercostal blocks with intravenous analgesia. The characteristics of the included studies are shown in Appendix Table 4.

### Epidural analgesia versus intravenous analgesia

**Fig. 2:**
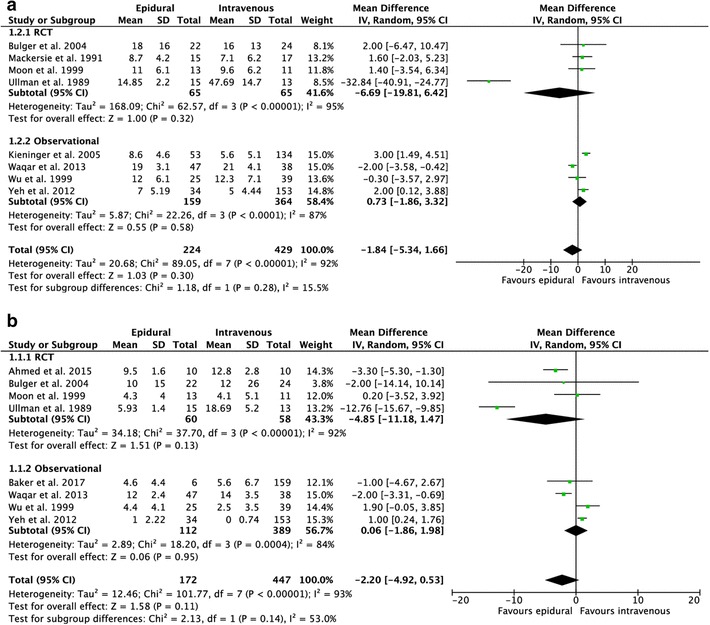

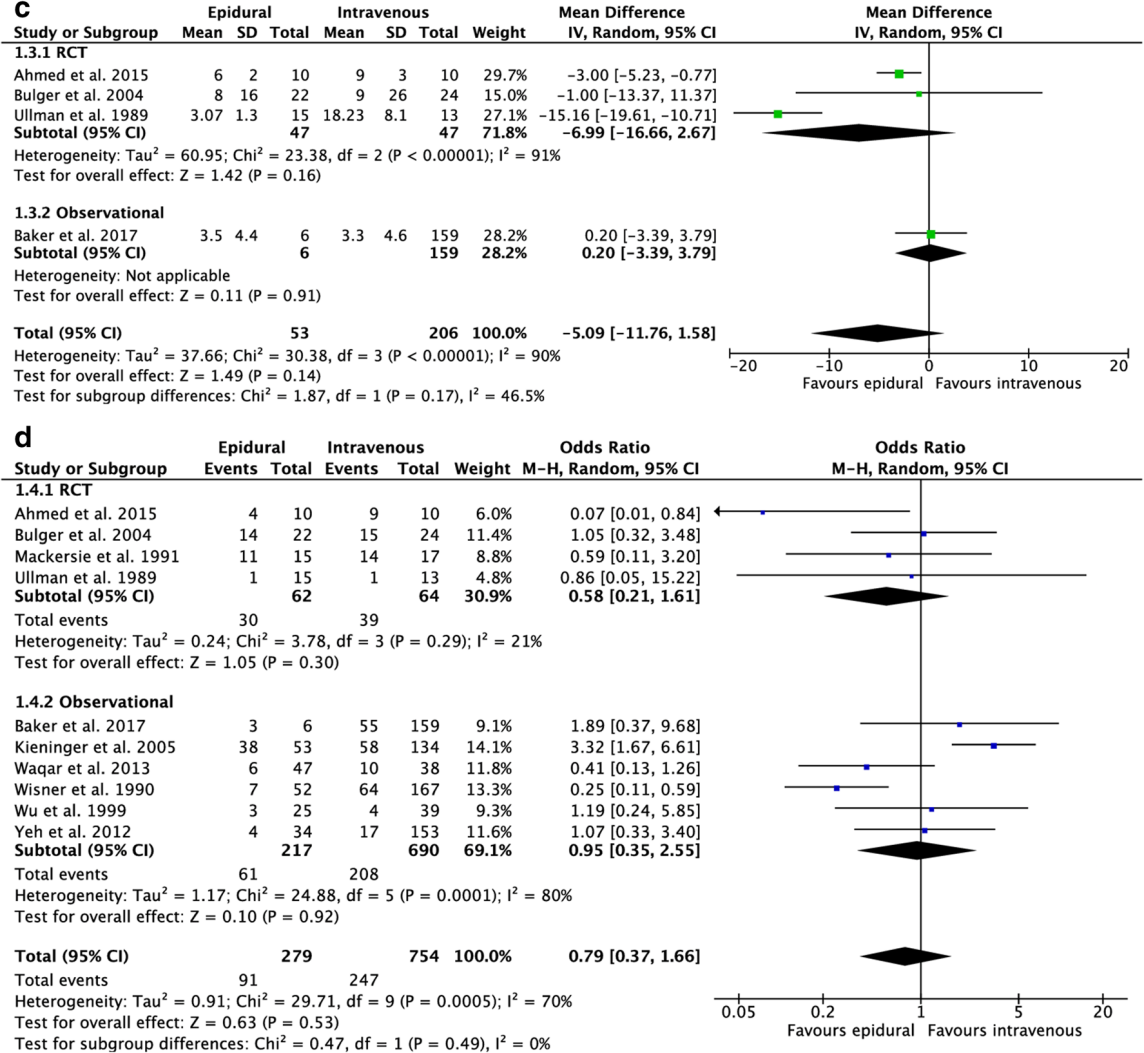
Forest plot of the length of **a** hospital stay **b** intensive care unit stay **c** mechanical ventilation (epidural vs intravenous). **d** forest plot of the pulmonary complications (epidural vs intravenous)

The results of the studies comparing epidural with intravenous analgesia are summarized in Appendix Table 5. Meta-analyses are shown in Fig. 2. Of the 11 included studies, 4 studies [16, 20, 21, 28] examined pain scores on different intervals after treatment with epidural or intravenous analgesia. One study [16] described lower pain scores at all intervals of the study period in the group that received epidural analgesia (*p* < 0.05). Significant lower pain scores on coughing were found in the first 24 h in the epidural group (*p* < 0.05). One study [20] found significantly lower pain scores at all intervals (*p* < 0.05), except on the baseline interval (*p* = 0.82), in the group that received epidural analgesia. One [28] study found significant differences (*p* < 0.05) in pain relief on day 1 and on day 3 in favor of the patients that received epidural analgesia, no differences were found on day two. One study [21] reported that the improvement in pain was more pronounced in the group that received epidural analgesia, but no significant difference was found between the two groups (*p* = 0.08). The results on pain relief are shown in Table 1.

**Table 1:** Results of pain relief

First author	Pain assessment tool	Outcome (mean ± SD)		
*Epidural analgesia vs intravenous analgesia*				
Waqar et al.	Verbal Rating Scale (0–5)	Significant lower pain scores at all intervals in epidural group (*p* < 0.05) Significant lower pain scores on coughing in the first 24 h in epidural group (*p* < 0.05)		
Wu et al.	Standardized form (0–5)^a^	Baseline After 8 h After 24 h After 48 h After 72 h	[4 (3, 4) vs 4 (3.3, 4), *p* < 0.82] [2 (2, 1) vs 3 (2, 4), *p* < 0.001] [1 (1, 2) vs 3 (3, 4), *p* < 0.001] [2 (1, 2) vs 3 (2, 3), *p* < 0.001] [1 (1, 2) vs 3 (2, 3), *p* < 0.001]	
*Moon et al.	Verbal Rating Scale (0–10)^b^	First 24 h After 48 h After 72 h	(5.8 vs 7.5, *p* < 0.05) (6.0 vs 6.3) (3.8 vs 6.2, *p* < 0.05)	
*Mackersie et al.	Visual Analogue Scale (0–100)^b^	Percentage change in VAS score		
		At rest	(− 32 ± 24 vs − 27 ± 27, *p* < 0.05)	
		Coughing and deep breathing	(− 42 ± 25 vs − 25 ± 26, *p* < 0.05)	
			At rest	Coughing
		Pre-analgesia Post-analgesia After 48 h After 72	(56 vs 62) (24 vs 37) (28 vs 38) (19 vs 26)	(88 vs 89) (45 vs 63) (51 vs 53) (42 vs 58)
*Epidural analgesia vs intercostal block*				
*Hashemzadeh et al	Verbal rating scale (0–10)	Mean pain score during hospital admission		
		At rest Coughing	(2.2 ± 0.74 vs 3.3 ± 1.005) (3.05 ± 0.88 vs 4.95 ± 0.99)	
Truitt et al	Numeric pain score (0–10)	Significant improvement of pain score after CINB catheter placement (*p* < 0.05)		
			At rest	Coughing
		Pre-analgesia Post-analgesia	(7.5) (2.6)	(9.4) (3.6)
		No comparison with epidural group		
*Epidural analgesia vs paravertebral block*				
Shapiro et al	Visual Analogue Scale (0–10)	Mean change in pain from admission to discharge: 3.0 vs 4.0 (*p* = 0.28)		
*Mohta et al	Visual Analogue Scale (0–100)^b^	No significant differences in mean VAS scores at rest (*p* = 0.426) and on coughing (*p* = 0.721)		
			At rest	Coughing
		Baseline After 0.5 h After 24 h After 72 h	(66 vs 66) (13 vs 13) (17 vs 7) (12 vs 9)	(97 vs 97) (31 vs 44) (42 vs 34) (32 vs 32)
*Intercostal block vs intravenous analgesia*				
Hwang et al	Visual Analogue Scale (0–10)	Baseline Post-analgesia After 24 h After 7 days	At rest	
			(9.43 vs 8.16) (5.39 vs 7.42, *p* = 0.007) (5.04 vs 6.16, *p* = 0.024) (3.65 vs 3.81, *p* = 0.944)	
*Paravertebral block vs intravenous analgesia*				
*Yeying et al	Visual Analogue Scale (0–10)		At rest	Coughing
		Baseline After 1 h After 24 h After 48 h After 72 h	(7.6 ± 2.2 vs 7.8 ± 2.1) (3.9 ± 1.3 vs 4.9 ± 1.5, *p* < 0.05) (3.4 ± 1.0 vs 4.1 ± 1.2, *p* < 0.05) (2.8 ± 0.9 vs 3.0 ± 1.0) (2.1 ± 0.5 vs 2.2 ± 0.6)	(7.9 ± 2.0 vs 8.0 ± 2.2) (4.5 ± 1.6 vs 5.6 ± 1.7, *p* < 0.05) (3.9 ± 1.1 vs 4.5 ± 1.3, *p* < 0.05) (3.3 ± 0.8 vs 3.5 ± 0.9, *p* < 0.05) (2.7 ± 0.6 vs 2.8 ± 0.7, *p* < 0.05)

*CINB* continuous intercostal nerve block, *h* hour, *SD* standard deviation, *VAS* visual Analogue scale, *vs* versus

^*^RCT

^a^Pain scores expressed as median (with 25th and 75th percentiles)

^b^Pain scores shown as estimated scores by reading of the figures

Eight studies reported on the length of hospital stay [8, 16, 18–21, 24, 28]. The average number of days of hospitalization was lower in the epidural group (12.4 ± 4.5) compared with the group that received intravenous analgesia (15.5 ± 14.1), pooled analysis failed to show statistical significance [95% CI, mean difference (MD) − 1.84 (− 5.34, 1.66), *I*^2^ = 92%, *p* = 0.30]. Eight studies reported on the length of ICU stay [8, 17–19, 21, 25, 28, 29;17–19;21;25;28;29]. The average number of days on the ICU was lower in the epidural group (6.4 ± 3.7) compared with the intravenous group (8.7 ± 6.5), again pooled analysis showed no significant differences [95% CI, MD − 2.20 (− 4.92, 0.53), *I*^2^ = 93% *p* = 0.11]. Five [8, 16, 17, 24, 27] studies reported on the duration of mechanical ventilation. Four [8, 17, 24, 27] studies were eligible for pooled analysis because the data of one study were not available. The average of days on mechanical ventilation was lower (5.2 ± 2.3) in the epidural group compared with the intravenous group (9.9 ± 6.2). Pooled analysis showed no significant differences between the groups [95% CI, MD − 5.09 (− 11.76, 1.58), *I*^2^ = 90%, *p* = 0.14].

Ten studies [8, 16–21, 24, 28, 29] reported on the occurrence of pulmonary complications. The number of pulmonary complications ranged from 10 to 90% and pooled analysis showed no significant differences [95% CI, OR 0.79 (0.37, 1.66), *I*^2^ = 70%, *p* = 0.53].

### Epidural analgesia versus intercostal block

The results of the studies comparing epidural analgesia with intercostal blocks are summarized in Appendix Table 6. Meta-analyses are shown in Appendix Fig. 3. As a consequence of insufficient data and variability of outcome measurement, meta-analyses were only possible for the length of hospital and ICU stay.

Two studies [22, 26] reported on pain scores. One study [26] described solely pain scores of the group that received intercostal blocks. Placement of the intercostal catheter resulted in significant improvement in pain severity (*p* < 0.05). No comparison was made with the historical control group that received epidural analgesia. According to one study [22], epidural analgesia provides better control of pain than the intercostal modality. The mean VAS scores that were observed during hospitalization were 2.2 ± 0.74 at rest and 3.05 ± 0.88 with cough in the epidural group, respectively 3.3 ± 1.01 and 4.95 ± 0.99 in the intercostal group.

Three studies [22, 25, 26] reported on the length of hospital stay. The average number of days of hospitalization was 7.1 ± 2.3 with epidural analgesia and 6.0 ± 2.7 with intercostal blocks. One study [26] was not included for pooled analysis because the standard deviations were not reported. Pooled analysis of the two remaining studies showed no significant differences [95% CI, MD − 0.13 (− 4.18, − 3.91), *I*^2^ = 81%, *p* = 0.95].

Two studies [22, 25] reported on the length of ICU stay, pooled analysis showed no significant differences [95% CI, MD − 0.37 (− 0.93, 0.19), *I*^2^ = 0%, *p* = 0.20].

### Epidural analgesia versus paravertebral block

The results of the studies comparing epidural analgesia with paravertebral blocks are summarized in Appendix Table 7. Meta-analyses are shown in Appendix Fig. 4. Two studies reported on pain scores. One study [6] found no significant intergroup difference in mean pain scores either at rest (*p* = *0.426)* or on coughing *(p* = *0.721)* on different intervals, and one study [30] described that there was no difference between both groups in the mean change of pain during hospital admission (Table 1).

Three studies [6, 30, 31] reported on the length of hospital and ICU stay. The average number of days of hospitalization was 8.3 ± 1.7 with epidural analgesia and 8.6 ± 2.6 with paravertebral blocks, respectively, 4.5 ± 2.1 and 4.6 ± 1.9 for the length of ICU stay. Pooled analysis showed no significant differences for the length of hospital stay [95% CI, MD 0.09 (− 0.45, 0.63), *I*^2^ = 1%, *p* = 0.74], respectively, for the length of ICU stay [MD − 0.08 (− 1.68, 1.52), *I*^2^ = 87%, *p* = 0.92].

### Intercostal block versus intravenous analgesia

One study [23] compared intravenous analgesia with intercostal blocks. The average number of hospital days and the VAS pain scores were reported, and are summarized in Appendix Table 8, respectively, Table 1. Significant differences in pain relief were described on different intervals, in favor of the intercostal blocks.

### Paravertebral block versus intravenous analgesia

One study [32] compared paravertebral blocks with intravenous analgesia. The mortality and the VAS pain scores were reported, and are summarized in Appendix Table 9, respectively Table 1. Significant differences in pain relief were described on different intervals, in favor of the paravertebral blocks.

### Sensitivity and subgroup analyses

The sensitivity and subgroup analyses are shown in Appendix Table 10. The results remained non-significant for all secondary outcomes in the group comparing epidural analgesia with intravenous analgesia and in the group comparing epidural analgesia with paravertebral blocks.

## Discussion

This systematic review and meta-analysis of both RCTs and cohort series focused on the analgesic therapy for patients with traumatic rib fractures. Results of this study show that overall epidural analgesia provides better pain relief than the other modalities. In three studies [16, 20, 28] significant differences (*p* < 0.05) were found in the improvement of pain in favor of epidural analgesia when compared with intravenous analgesia. In one study [21], the reduction of pain appeared to be more definite in the group that received epidural analgesia.

With respect to the secondary outcomes, our systematic review and meta-analysis failed to show significant differences between the analgesic modalities. Most of these outcome parameters are multifactorial and heterogeneously determined. Therefore, the relationship between the intervention and the secondary outcome parameters is influenced by multiple underlying factors, other than the type of analgesia. To alleviate the influence of these factors, heterogeneity corrections and sensitivity analyses were conducted. As a result, the trends that were initially observed in the group comparing epidural analgesia with intravenous analgesia for length of ICU stay (*p* = 0.11) and length of mechanical ventilation (*p* = 0.14), were not consistent after excluding outlier studies [24].

A recent systematic review and meta-analysis on this subject by Duch et al. [10], found a significant increased intervention effect for the reduction of pain, in favor of epidural analgesia, when compared with the paravertebral or intercostal modality. Because these results were based on only two studies and no significant differences were found on the other outcomes, they concluded that there was no firm evidence to assume that epidural analgesia has advantages over the other modalities. Likewise, a systematic review of 2008 from Carrier et al. [11], reported that there was no improvement in mortality, length of hospital and ICU stay, or duration of mechanical ventilation, if epidural analgesia was compared with other analgesic interventions. Our results differ from theirs in several aspects. Most importantly, our study showed that there is evidence that epidural analgesia results in better pain relief than the other modalities. The results of our secondary outcomes are in accordance with the aforementioned reviews, and seem to rely on a multifactorial basis. In contrast to the studies of Duch et al. [10] and Carrier et al. [11], we included observational studies. Therefore, we were able to include several (new) studies [16–20, 23, 25–27, 29–32] resulting in a larger patient database.

The current guideline of the Eastern Association for the Surgery of Trauma (EAST) recommend epidural analgesia or a multimodal approach over opioids alone, for pain relief in patients with blunt chest trauma [9]. In comparison with this guideline of the EAST, our study differs in certain respects. First, a major distinction is that in our study, the results of the single modalities were separately compared with each other. In the guideline of the EAST, the single modalities were compared with the merged results of larger groups. The epidural, paravertebral and intercostal modalities were in particular compared with the results of patients receiving “non regional’’ analgesia, and the interpleural modality was compared with “other regional modalities’’. Analysis to demonstrate the differences between the single modalities were not implemented. Second, four studies [4, 47, 49, 50] using mixed cohorts of patients, in which the analgesic interventions used in the control group were not extractable, were also excluded in our study. Third, we were able to include six new studies [16, 17, 27, 30–32].

A potential advantage of our method is that by comparing the single analgesic interventions, subtle differences might be more accurately ascertainable. Besides, because the studies were compared separately, our method and results might approach closer to reality.

Another strength of this systematic review is that a considerable amount of extra studies was included due to inclusion of observational studies. In addition, as stated in recently published systematic reviews [15, 51, 52], the inclusion of both RCTs and observational studies might lead to more study power. If observational studies are of sufficient quality, the results will correspond with those of an RCT [15, 51, 52]. Furthermore, it appears to give a better reflection of common clinical practice, which might improve the generalizability and applicability of the outcomes of a systematic review [51, 52].

On the other hand, the included studies were of low methodological quality, as assessed using the MINORS score. Therefore, the overall quality and applicability of the available evidence is low, and there is potentially a high risk of bias. Besides, merely a small amount of studies investigated the management of pain. Of the studies reporting on pain, patient samples were overall small, outcome measurements varied and exact pain scores were often not or poorly reported. Pooled analyses for pain in patients with traumatic rib fractures were not feasible due to inadequate reported data. Conversion of pain scores to one comprehensive score was not performed due to increase of bias. Furthermore, the studies were overall difficult to compare because of the heterogeneity in the study method and investigated endpoints. Analgesia-related complications such as nausea, vomiting, catheter inflammation, hypotension, respiratory depression, itching and rash, were also not frequently reported. However, pulmonary complications, which are considered to be important complications in patients with traumatic rib fractures, where in general adequately reported and could be properly investigated. As described in the results, there were no significant differences in the occurrence of pulmonary complications between the three analgesic therapies.

Pooled analyses between epidural and paravertebral was for a greater part determined by the large sample size of Malekpour et al. [31]. As we could only include three studies in these analyses, this might have influenced the outcome.

The value of the different analgesic modalities in critical care patients is insufficiently described. Only one of our included studies compared epidural analgesia with parenteral analgesia in mechanically ventilated ICU patients with flail chest [17]. This RCT described a significant difference in the length of ICU stay, the duration of mechanical ventilation and the change in tidal volume in the first 24 h of ICU admission, in favor of epidural analgesia.

The type of medication is not reflected in our analysis. The different modalities were compared, as described in the baseline characteristics (Appendix Table 4). However, it could be relevant if only opioids were administered, or if local anesthetics were also applied. Furthermore, there was insufficient information about any additional pain medication and whether escape medication was prescribed.

Although there seemed to be significant differences between the different analgesic therapies, further research on the analgesic therapy for traumatic rib fractures is desirable to extend our knowledge of the reduction of pain. Many different pain assessment tools are used in the current practice. The NRS pain score at breathing/coughing seems to be the most reliable outcome parameter, since it reflects the influence of pain on function of the ribcage. To compare the results of pain reduction more homogeneously, future studies should use a universal pain assessment tool. Second, besides pain measurement, there should also be data available on the use of other multimodal treatments started, the daily total opioid consumption and efficacy of the interventional analgesic therapy. On account of the increasing contraindications and the high probability of failure of the epidurals, research into safe and effective pain management by other analgesic methods must be continued.

Another future perspective is to determine the contribution of surgical rib fixation for the primary and secondary outcomes as described in this systematic review.

## Conclusion

Results of this study show that epidural analgesia provides better pain relief than the other modalities. No differences were observed for secondary endpoints like length of ICU stay, length of mechanical ventilation or pulmonary complications. However, the quality of the available evidence is low, and therefore, preclude strong recommendations.

## Figures and Tables

**Fig. 3:**
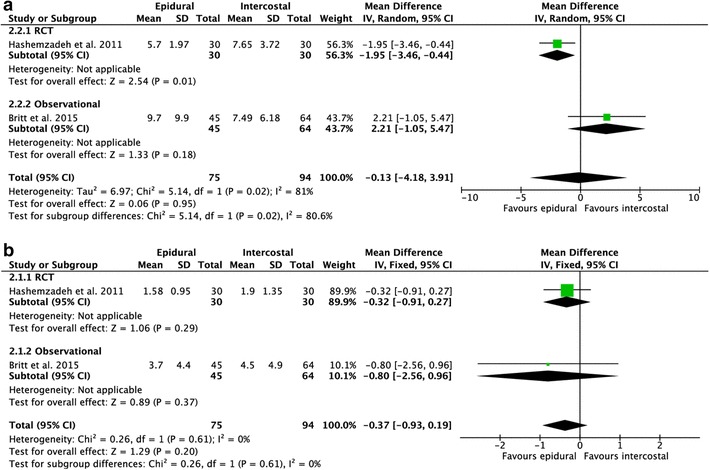
Forest plot of the length of **a** hospital stay **b** intensive care unit stay (epidural vs intercostal)

**Fig. 4:**
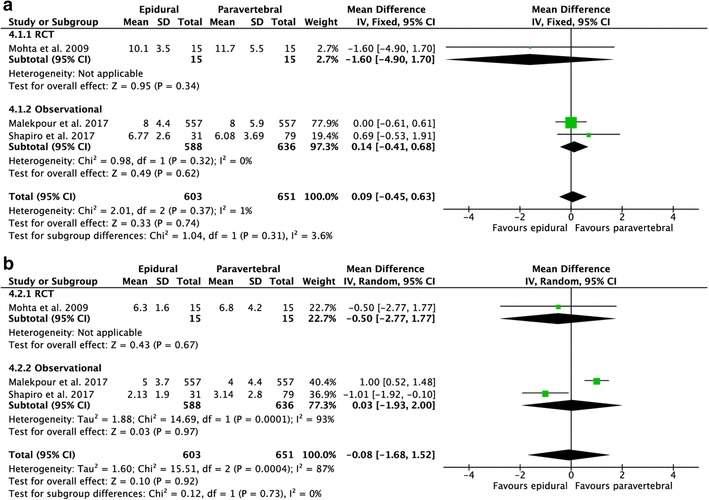
Forest plot of the length of **a** hospital stay **b** intensive care unit stay (epidural vs paravertebral)

## Appendix

See Tables (2, 3, 4, 5, 6, 7, 8, 9, 10) and Figs. (3, 4).

**Table 2:** Search syntax representing the used search strings in the different databases

Database	Search string	Hits
PubMed	(((((fracture[Title/Abstract] OR fractured[Title/Abstract] OR fractures[Title/Abstract]) AND (“Ribs“[Mesh] OR rib[Title/Abstract] OR ribs[Title/Abstract])))) OR “Rib Fractures“[Mesh]) AND ((((epidural[Title/Abstract] OR intercostal[Title/Abstract] OR interpleural[Title/Abstract] OR paravertebral[Title/Abstract] OR intrathecal[Title/Abstract] OR oral[Title/Abstract] OR parenteral[Title/Abstract]) AND (anesthesia[Title/Abstract] OR anesthesia[Title/Abstract] OR analgesia[Title/Abstract] OR block[Title/Abstract] OR blocks[Title/Abstract]) OR analgesics[Title/Abstract])) OR (“Pain“[Mesh] OR ((pain[Title/Abstract] OR pains[Title/Abstract]) AND (manag*[Title/Abstract] OR alleviat*[Title/Abstract] OR control*[Title/Abstract] OR reduc*[Title/Abstract] OR treat* OR therap*[Title/Abstract] OR scor*[Title/Abstract]))))	708
EMBASE	fracture:ab,ti OR fractures:ab,ti OR fractured:ab,ti AND (rib:ab,ti OR ‘rib’/exp OR ‘rib fracture’/exp OR ‘rib fracture’:ab,ti OR ribs:ab,ti) AND (epidural:ab,ti OR intercostal:ab,ti OR interpleural:ab,ti OR paravertebral:ab,ti OR intrathecal:ab,ti OR oral:ab,ti OR parenteral:ab,ti) AND (anesthesia:ab,ti OR anesthesia:ab,ti OR analgesia:ab,ti OR analgesics ab,ti OR block:ab,ti OR blocks:ab,ti OR ‘anaesthesia’/exp OR ‘epidural anesthesia’ OR ‘intravenous regional anesthesia’/exp OR ‘intercostal nerve block’/exp)	238
CENTRAL	Rib fracture	183

**Table 3:** Quality assessment of the included studies using the methodological index for non-randomized studies

MINORS	Baker et al.	Ahmed et al.	Waqar et al.	Yeh et al.	Kieninger et al.	Bulger et al.	Wu et al.	Moon et al.	Mackersie et al.	Wisner et al.	Ullman et al.	Britt et al.	Hashemzadeh et al.	Truitt et al.	Shapiro et al.	Malekpour	Mohta et al.	Yeying et al.	Hwang et al.
A clearly stated aim*	2	2	1	2	2	2	1	2	2	2	2	1	2	2	2	2	2	2	2
Inclusion of consecutive patients	1	0	0	2	1	1	2	1	0	0	2	2	2	2	0	0	0	2	0
Prospective collection of data	0	2	0	0	0	0	0	2	2	0	0	0	2	2	0	0	2	2	0
Endpoints appropriate to the aim of study	2	2	1	2	2	2	2	2	2	2	2	2	2	2	2	2	2	2	2
Unbiased assessment of the study endpoint	0	0	1	0	0	1	1	0	0	0	0	0	0	0	0	0	1	1	0
Follow-up period appropriate to the aim of the study**	1	1	1	1	1	1	1	1	1	1	1	1	1	1	2	1	1	2	1
Loss to follow-up less than 5%	2	2	0	2	2	2	2	1	2	0	2	2	2	2	0	0	2	2	2
Prospective calculation of the study size	0	0	0	0	0	1	0	0	0	0	0	2	0	0	0	1	0	2	0
Adequate control group	2	2	2	2	2	2	2	2	2	2	2	2	2	2	2	2	2	2	2
Contemporary groups	2	2	2	2	2	2	2	2	2	2	2	2	2	1	0	2	2	2	2
Baseline equivalence of groups	1	2	1	1	1	1	1	2	1	1	1	1	1	2	2	2	2	2	0
Adequate statistical analyses	2	2	2	2	2	2	2	2	2	2	2	2	2	2	1	2	2	2	2
Total MINORS score	15	17	11	16	15	17	16	17	16	12	16	17	18	18	11	14	18	23	13

The items are scored 0 (not reported), 1 (reported but inadequate), or 2 (reported and adequate). Additional criteria are established for the following points:

*A clearly stated aim: 2 points if described according to the PICO model for clinical questions [48], 1 point if one of the PICO criteria has not been satisfied, 0 points if not reported according to the PICO model

**Follow-up period: 2 points if follow-up > 6 weeks after hospitalization, 1 point if patients only were reviewed during hospitalization period, 0 points if not reported

**Table 4:** Baseline characteristics

First author, year of publication	Country	Design, setting	Patient characteristics		Intervention	Comparator	Number of patients		Male, *n* (%)		Age (mean ± SD)		ISS (mean ± SD)	
			Inclusion criteria	Exclusion criteria			INT	COM	INT	COM	INT	COM	INT	COM
*Epidural analgesia vs intravenous analgesia*														
Baker et al. 2016	UK	R, Level I trauma center	≥ 16 years ≥ 1 thoracic fractures (ribs, sternum, scapular and clavicular fractures)	Patients who died within 24 h of admission to hospital and patients with penetrating injuries	Continuous epidural analgesia, containing bupivacaine and fentanyl	Intravenous analgesia, morphine delivered by PCA	6	159	4 (66.7%)	122(76.7%)	65.9 ± 18.4	46.5 ± 17.8	25.3 ± 10.5	24.1 ± 10.5
Ahmed et al. 2015	India	RCT, ICU	18–55 years ≥ 3 rib fractures with flail segment required mechanical ventilation	Acute spine fracture, pre-existing spine deformity, severe traumatic brain or spinal cord injury, unstable pelvic fracture or open abdomen, ongoing cardiac instability or coagulopathy, and active chest wall infection	Thoracic epidural analgesia, 4 mL of 0.125% bupivacaine bolus followed by 4 mL/h of 2 µg/kg fentanyl as adjuvant	Intravenous analgesia, fentanyl 2 µg/kg	10	10	7(70%)	8(80%)	39.8 ± 8.8	36.7 ± 10.6	25 ± 7	28 ± 7
Waqar et al. 2013	Paki-stan	R, Surgical ICU	> 18 years ≥ 3 rib fractures	Contraindications to epidural catheter, pregnancy, allergy to local anesthetics or opioids, and associated injuries like intracranial hematoma	Thoracic epidural analgesia, bupivacaine	Intravenous opioid analgesia	47	38	35 (75%)	29 (76%)	54 ± 17	45 ± 22	23.6 ± 10.3	21.0 ± 6.7
Yeh et al. 2012	USA	R, Trauma service	> 18 years ≥ 3 rib fractures	Contraindications to epidural catheter, acute spine fractures or pre-existing spine deformity, traumatic brain injury or altered mental status or spinal cord injury, unstable pelvic fracture or open abdomen, hemodynamic instability and coagulopathies	Epidural analgesia, containing bupivacaine and fentanyl	Oral or intravenous narcotics, delivered by PCA	34	153	26(76.5%)	113(73.9%)	51.4 ± 15.0	48.8 ± 18.4	22.5 ± 8.2	22.6 ± 9.6
Kieninger et al. 2005	USA	R, Level I trauma center	> 55 years ≥ 1 rib fracture ISS score < 16	Sternal fracture, required intubation before admission to the trauma service or associated injuries that included intracranial hemorrhage	Epidural analgesia	Intravenous opioids	53	134	18(33.9%)	52(38.8%)	77.7 ± 10.2	77.3 ± 10.5	10.3 ± 3.6	8.3 ± 3.9
Bulger et al. 2004	USA	RCT, Level I trauma center	> 18 years ≥ 3 rib fractures	Acute spine fracture or pre-existing spine deformity, severe traumatic brain or spinal cord injury, or severe altered mental status, unstable pelvic fracture or open abdomen, active chest wall infection, and acute thoracic aortic transection	Thoracic epidural analgesia, bupivacaine, morphine and fentanyl	Intravenous opioid analgesia, morphine and fentanyl by PCA for alert patients and with nurse assistance for patients who could not participate in self-administration	22	24	17(77%)	16(67%)	49 ± 18	46 ± 16	26 ± 8	25 ± 8
Wu et al. 1999	USA	R, NR	> 18 years ≥ 3 rib fractures Following motor vehicle crash	NR	Thoracic epidural analgesia, 0.125 to 0.25% bupivacaine and 2.5 µg/kg fentanyl	Intravenous morphine, delivered by PCA	25	39	13(52%)	20(51%)	56 ± 17	45 ± 22	21.6 ± 10.3	21.9 ± 6.7
Moon et al. 1999	USA	RCT, NR	18–60 years > 3 consecutive rib fractures or a flail chest segment or pulmonary contusion or sternal fracture	Contraindications to epidural catheter placement (coagulopathy, infection at insertion site, sepsis, or hypovolemic shock), morbid obesity, evidence of spinal cord injury, GCS < 15, adrenal insufficiency, use of steroids, need for vasoactive agents to support blood pressure, immunodeficiency disease, pregnancy, inability to communicate effectively, or history of allergy to local anesthetics or opioids	Thoracic epidural analgesia, initial bolus of fentanyl 50 µg and morphine 3 mg followed by continuous infusion of bupivacaine 0.25% and morphine 0.005%, at a rate of 4 to 6 ml/hr	Intravenous analgesia, intravenous morphine 0.1 mg/kg loading doses followed by morphine 1 mg/ml delivered by PCA in bolus doses of 2 mg	13	11	8(61.5%)	6(54.5%)	37 ± NR	40 ± NR	26.6 ± NR	23.4 ± NR
Mackersie et al.1991	USA	RCT, Level I trauma center	> 18 years ≥ 3 rib fractures and flail chest or flail sternum or ≥ 2 rib fractures and exploratory laparotomy or pulmonary contusion	Pregnancy, history of substance abuse, psychiatric disorder, axial spine injury, chronic pain or chronic us of analgesics, and painful extremity injury	Continuous epidural analgesia, fentanyl bolus 1.0 µg/kg followed by continuous administration at an initial rate of 0.5 mg/kg/hour	Continuous intravenous,fentanyl bolus 5 µg/cc followed by continuous administration at an initial rate of 0.5 mg/kg/hour	15	17	NR	NR	49.3 ± 19	47.8 ± 14	20 ± 7.6	16.0 ± 7.2
Wisner et al.1990	USA	R, NR	≥ 60 Admission diagnosis of either rib fracture or sternal fracture	NR	Epidural analgesia, morphine sulfate bolus or continuous infusions of fentanyl	Intravenous or intramuscular	52	167	22(42.3%)	74(44.3%)	71.0 ± 1.1	69.4 ± 0.6	15.7 ± 1.0	14.6 ± 0.8
Ullman et al.1989	USA	RCT, Surgical ICU	≥ 3 unilateral fractured ribs or flail segment with significant contusion of the chest wall with impaired ventilation	NR	Thoracic epidural analgesia, loading dose fentanyl 100 µg with morphine 5 mg, and continuous morphine 70 µg/ml	Continuous intravenous morphine	15	13	11(73.3%)	11(84.6%)	46.1 ± 4.6	53.0 ± 6.0	19.5 ± 2.03	25.3 ± 2.9
*Epidural analgesia vs intercostal block*														
Britt et al. 2015	USA	R, Level II trauma center	> 18 years ≥ 2 rib fractures	NR	Epidural analgesia, bupivacaine 0.1% with 5 µg/mL fentanyl	Continuous intercostal nerve block, bupivacaine 0.5% continuous 4 mL/hour	45	64	31(68.9%)	38(58.5%)	60.9 ± 17.3	70.5 ± 6.9	13.6 ± 5.2	12.5 ± 6.2
Hashemzadeh et al. 2011	Iran	RCT, ICU	> 18 years > 1 rib fracture GCS > 14	Liver or blunt splenic trauma, decreased consciousness, cerebral injury, mechanical ventilation, coagulopathy, fever and systemic or epidural infection	Thoracic epidural analgesia, bupivacaine 0.125 and 1 mg morphine every 8 h, and pethidine 0.5 ml PRN	Intercostal nerve block, bupivacaine 0.25% every 8 h, and pethidine 0.5 ml PRN	30	30	28(95%)	27(90%)	45.5 ± 15.4	64.5 ± 7.2	NR	NR
Truitt et al. 2011	USA	P, NR	> 18 years ≥ 3 unilateral rib fractures	Intubated before CINB placement, confounding injuries (traumatic brain injury, pelvic fracture, and long bone fracture), and allergy to anesthetics	Continuous intercostal nerve block	Epidural analgesia	102	75	NR	NR	69	68	14	15
*Epidural analgesia vs paravertebral block*														
Shapiro et al. 2017	USA	R, Level II trauma center	≥ 2 unilateral rib fractures	Bilateral rib fractures	Epidural analgesia	Paravertebral analgesia, bupivacaine 0.5%	31	79	NR	NR	61.4 ± 18.1	68.7 ± 18.1	NR	NR
Malekpour et al. 2017^a^	USA	R, NR	> 18 years > 1 rib fracture	Patients with sternum, larynx, and trachea fractures	Epidural analgesia	Paravertebral block	1073	1110	740 (69%)	706 (63.9%)	58 ± 16.3	54.5 + 17.8	17 (11–22)	14 (10–22)
Mohta et al. 2009	India	RCT, NR	> 18 years ≥ 3 unilateral rib fractures	Unconscious patients, unstable cardiac status or severely altered mental status, liver or kidney disease, contraindications to TEA or TPVB, pre-existing spinal deformity, use of anticoagulants or coagulopathy	Continuous thoracic epidural	Thoracic paravertebral	15	15	12 (80%)	12(80%)	38.9 ± 14.9	40.4 ± 14.8	15.9 ± 7.1	13.6 ± 5.6
*Paravertebral block vs intravenous analgesia*														
Yeying et al. 2017	China	RCT, Level I trauma center	≥ 18 years ≥ 3 unilateral rib fractures	Age < 18 or > 70, severe head injury or unconsciousness, pathological obesity (BMI ≥ 35), thoracic and abdominal visceral injuries, unstable cardiac status, severe liver or kidney disease, coagulopathy, spinal or pelvic fracture, infection at the puncture site and allergy to local anesthetics	Paravertebral block, 250 ml 0.2% ropivacaine 5 mL/h, with a 5 ml bolus dose, and lockout interval of 15 min	Intravenous analgesia, 100 ml 2 µg/kg sufentanil (diluted with saline) 2 ml/h, with a 2 ml bolus dose, and lockout interval of 15 min	45	45	29 (64.4%)	68.9%	39.1 ± 8.9	41.2 ± 9.7	14.2 ± 5.1	13.7 ± 5.5
*Intercostal block vs intravenous analgesia*														
Hwang et al. 2014	Korea	R, NR	≥ 1 rib fracture	NR	Conventional (iv PCA and/or fentanyl patch) + continuous intercostal nerve block (CINB)	Conventional pain control (iv PCA and/or fentanyl patch)	23	31	44 (81.4%)		48.5 ± NR		NR	NR

*CINB* continuous intercostal nerve block, *COM* comparator group, *GCS* Glasgow coma score, *ICU* intensive care unit, *INT* intervention group, *ISS* injury severity score, *NR* not reported, *PCA* patient-controlled analgesia, *PRN* pro re nata, *P* prospective cohort study, *RCT* randomized controlled trial, *R* retrospective, *SD* standard deviation, *TEA* thoracic epidural analgesia, *TPVB* thoracic paravertebral block; UK, United Kingdom; USA, United States of America.

^a^Patient characteristics before propensity matching

**Table 5:** Results of studies comparing epidural analgesia with intravenous analgesia

First author	Number of patients		Mortality (during hospital admission)		Mechanical ventilation (days)		Hospital LOS (days)		Length of ICU stay (days)		Pulmonary complications		Other complications	
	EPI	IV	EPI	IV	EPI	IV	EPI	IV	EPI	IV	EPI	IV	EPI	IV
Baker et al.	6	159	0 (0%)	1 (16.7%)	3.5 ± 4.4	3.3 ± 4.6	17.6 ± 22.6^a^		4.6 ± 4.4	5.6 ± 6.7	Pneumonia *n* = 3 (50%) Respiratory tract infection *n* = 1 (16.7%)	Pneumonia *n* = 55 (34.6%) Respiratory tract infection *n* = 12 (7.5%)	NR	NR
Ahmed et al.	10	10	0 (0%)	1 (10%)	6 ± 2	9 ± 3	NR	NR	9.5 ± 1.6	12.8 ± 2.8	Pneumonia *n* = 2 (20%) ARDS *n* = 2 (20%)	Pneumonia *n* = 4 (40%) ARDS *n* = 5 (50%)	Hypotension *n* = 2 (20%) Bradycardia *n* = 1 (10%)	Hypotension *n* = 0 (0%) Bradycardia *n* = 0 (0%)
Waqar et al.	47	38	2 (4%)	1 (2.6%)	Reduction of days in epidural group		19 ± 3.1	21 ± 4.1	12 ± 2.4	14 ± 3.5	Pneumonia *n* = 6 (13%)	Pneumonia *n* = 10 (26%)	Cardiac *n* = 2 (4%)	Cardiac *n* = 1 (2.6%)
Yeh et al.	34	153	NR	NR	NR	NR	7 (5–12)^b^	5 (4–10)^b^	1 (0–3)^b^	0 (0–1)^b^	Overall *n* = 4 (11.8%)	Overall *n* = 17 (11%)	Overall *n* = 7 (20.6%)	Overall *n* = 25 (16.3%)
Kieninger et al.	53	134	5 (2.6%)		NR	NR	8.6 ± 4.6	5.6 ± 5.1	NR	NR	Overall *n* = 38 (72%)	Overall *n* = 58 (43%)	NR	NR
Bulger et al.	22	24	2 (9%)	1 (4.2%)	8 ± 16	9 ± 26	18 ± 16	16 ± 13	10 ± 15	12 ± 26	Pneumonia *n* = 4 (18%) ARDS *n* = 10 (45%)	Pneumonia *n* = 9 (38%) ARDS *n* = 6 (25%)	Pruritus *n* = 5 (27%) Transient motor block *n* = 2 (9%) Catheter site inflammation or superficial infection *n* = 1 (5%) Hypotension *n* = 1 (5%)	Pruritus *n* = 5 (21%) Nausea/vomiting *n* = 6 (25%) Depressed level of consciousness *n* = 1 (4%)
Wu et al.	25	39	0 (0%)	0 (0%)	NR	NR	12.0 ± 6.1	12.3 ± 7.1	4.4 ± 4.1	2.5 ± 3.5	Pneumonia *n* = 3 (12%)	Pneumonia *n* = 4 (10%)	Cardiac *n* = 1 (4%) Neurologic *n* = 1 (4%)	Cardiac *n* = 5 (13%) Neurologic *n* = 7 (18%)
Moon et al.	13	11	0 (0%)	0 (0%)	NR	NR	11 ± 6.1	9.6 ± 6.2	4.3 ± 4.0	4.1 ± 5.1	NR	NR	NR	NR
Mackersie et al.	15	17	0 (0%)	0 (0%)	NR	NR	8.7 ± 4.2	7.1 ± 6.2	NR	NR	Pneumonia *n* = 0 (0%) Atelectasis *n* = 11 (73%)	Pneumonia *n* = 0 (0%) Atelectasis *n* = 14 (82%)	Nausea/vomiting *n* = 7 (46%) Itching/rash *n* = 2 (13%)	Nausea/vomiting *n* = 5 (29%) Itching/rash *n* = 4 (23%)
Wisner et al.	52	167	2 (4%)	26 (16%)	4.4 ± 0.7		NR	NR	NR	NR	Pneumonia *n* = 4 (8%) ARDS *n* = 3 (6%) Effusion *n* = 0 (0%) Pneumothorax *n* = 0 (0%) Lung collapse *n* = 0 (0%)	Pneumonia *n* = 32 (19%) ARDS *n* = 24 (14%) Effusion *n* = 2 (1%) Pneumothorax *n* = 2 (1%) Lung collapse *n* = 4 (2%)	Major complications *n* = 0 (0%) Delayed respiratory depression *n* = 0 (0%) Erythema at catheter site *n* = 2 (4%) Urinary retention *n* = 0 (0%)	NR
Ullman et al.	15	13	NR	NR	3.1 ± 1.3	18.2 ± 8.1	14.9 ± 2.2	47.7 ± 14.7	5.9 ± 1.4	18.7 ± 5.2	None	None	Urinary retention *n* = 2 (13.3%)	None

*ARDS* acute respiratory distress syndrome, *EPI* epidural group, *IV* intravenous group, *LOS* length of stay, *NR* not reported

^a^Average of all studied groups, including patients receiving epidural analgesia, PCA, combination of epidural and PCA, and interval administered analgesia (included oral, intramuscular, subcutaneous and narcotic agents given intermittently or Pro Re Nata)

^b^Data presented as median (interquartile range)

**Table 6:** Results of studies comparing epidural analgesia with intercostal block

First author	Number of patients		Mortality		Mechanical ventilation (days)		Hospital LOS (days)		Length of ICU stay (days)		Pulmonary complications		Other complications	
	EPI	IB	EPI	IB	EPI	IB	EPI	IB	EPI	IB	EPI	IB	EPI	IB
Britt et al.	45	64	NR	NR	No significant intergroup difference in ventilator days (*p* = 0.61)		9.7 ± 9.9	7.5 ± 6.2^a^	3.7 ± 4.4	4.5 ± 4.9	Pneumonia or ventilator-dependent respiratory failure *n* = 8 (12.5%)	Pneumonia or ventilator-dependent respiratory failure *n* = 8 (12.5%)	NR	NR
Hashemzadeh et al.	30	30	NR	NR	NR	NR	5.7 ± 2.0	7.7 ± 3.7	1.6 ± 1.0	1.9 ± 1.4	No intergroup difference regarding incidence of respiratory complications		NR	NR
Truitt et al.	75	102	NR	NR	NR	NR	5.9	2.9	NR	NR	NR	NR	NR	NR

*EPI* epidural group, *IB* intercostal block group, *ICU* intensive care unit, *LOS* length of hospital stay

^a^Includes outlier

^b^No comparison with historical epidural control group

**Table 7:** Results of studies comparing epidural analgesia with paravertebral block

First author	Number of patients		Mortality		Mechanical ventilation (days)		Hospital LOS (days)		Length of ICU stay (days)		Pulmonary complications		Other complications	
	EPI	PVB	EPI	PVB	EPI	PVB	EPI	PVB	EPI	PVB	EPI	PVB	EPI	PVB
Shapiro et al.	31	79	0 (0%)	0 (0%)	NR	NR	6.77 ± 2.6	6.08 ± 3.69	2.13 ± 1.9	3.14 ± 2.8	NR	NR	0 (0%)	0 (0%)
Malekpour et al.	557	557	8 (1.4%)	12 (2.2%)	4 ± 4.4	5 ± 6.6	8 ± 4.4	8 ± 5.9	5 ± 3.7	4 ± 4.4	Pneumonia *n* = 40 (7.2%)	Pneumonia *n* = 40 (7.2%)	NR	NR
Mohta et al.	15	15	0 (0%)	0 (0%)	NR	NR	10.1 ± 3.5	11.7 ± 5.5	6.3 ± 1.6	6.8 ± 4.2	Pneumonia *n* = 1 (6.7%) Delayed pleural effusion *n* = 1 (6.7%)	Pneumonia *n* = 2 (13.3%) Delayed pleural effusion *n* = 0 (0%)	Hypotension *n* = 6 (40%)	Hypotension *n* = 2 (13.3%)

*EPI* epidural group, *PVB* paravertebral group, *ICU* intensive care unit, *LOS* length of hospital stay, *NR* not reported

**Table 8:** Results of studies comparing intercostal block with intravenous analgesia

First author	Number of patients		Mortality		Mechanical ventilation (days)		Hospital LOS (days)		Length of ICU stay (days)		Pulmonary complications		Other complications	
	IB	IV	IB	IV	IB	IV	IB	IV	IB	IV	IB	IV	IB	IV
Hwang et al.	23	31	NR	NR	NR	NR	9.35 (2–49)	10.61 (4–22)	NR	NR	0 (0%)	0 (0%)	NR	NR

*IB* intercostal block group, *IV* intravenous group, *ICU* intensive care unit, *LOS* length of hospital stay, *NR* not reported

**Table 9:** Results of studies comparing paravertebral block with intravenous analgesia

First author	Number of patients		Mortality		Mechanical ventilation (days)		Hospital LOS (days)		Length of ICU stay (days)		Pulmonary complications		Other complications	
	PVB	IV	PVB	IV	PVB	IV	PVB	IV	PVB	IV	PVB	IV	PVB	IV
Yeying et al.	45	45	0 (0%)	0 (0%)	NR	NR	NR	NR	NR	NR	3 (6.7%)	9 (20%)	Nausea/vomiting *n* = 3 (6.7%)	Nausea/vomiting *n* = 13 (28.9%)

*EPI* epidural group, *ICU* intensive care unit, *LOS* length of hospital stay, *NR* not reported, *PVB* paravertebral group

**Table 10:** Results of sensitivity and subgroup analysis

Comparison	Outcome	Results	Sensitivity analyses on study design	Sensitivity analyses on study quality	Sensitivity analyses on time	Sensitivity analyses on outlier studies	Subgroup analyses on etiology
Epidural analgesia vs intravenous analgesia	Hospital LOS*	− 1.84 (− 5.34; 1.66)	− 6.69 (− 19.81; 6.42)	− 6.99 (− 16.66; 2.67)	1.08 (− 1.82; 3.98)	0.97 (− 0.98; 2.91)	− 2.33 (− 6.16; 1.49)
	Length of ICU stay*	− 2.20 (− 4.92; 0.53)	− 4.85 (− 11.18; 1.47)	***	− 1.28 (− 3.50; 0.95)	− 0.55 (− 2.27; 1.18)	− 2.79 (− 6.09; 0.52)
	Mechanical ventilation*	− 5.18 (− 11.77; 1.42)	− 6.99 (− 16.66; 2.67)	− 2.15 (− 4.60; 0.30)	− 1.96 (− 4.09; 0.18)	− 1.96 (− 4.09; 0.18)	− 5.18 (− 11.77; 1.42)
	Pulmonary complications**	0.79 (0.37; 1.66)	0.58 (0.21; 1.61)	0.35 (0.03; 4.56)	0.97 (0.39; 2.44)	****	0.89 (0.41; 1.92)
Epidural analgesia vs paravertebral blocks	Hospital LOS*	0.09 (− 0.45; 0.63)	***	− 0.05 (− 0.65; 0.55)	0.14 (− 0.41; 0.68)	****	***
	Length of ICU stay*	− 0.08 (− 1.68; 1.52)	***	0.68 (− 0.53; 1.88)	0.03 (− 1.93;2.00)	****	***

*Results are presented as mean difference (95%CI)

**Results are presented as odds ratio (95%CI)

***Analysis not performed because < one study can be included

****Analysis not performed because no outlier studies present
